# Asymptomatic Helminth Infection in Active Tuberculosis Is Associated with Increased Regulatory and Th-2 Responses and a Lower Sputum Smear Positivity

**DOI:** 10.1371/journal.pntd.0003994

**Published:** 2015-08-06

**Authors:** Ebba Abate, Meseret Belayneh, Jonna Idh, Ermias Diro, Daniel Elias, Sven Britton, Abraham Aseffa, Olle Stendahl, Thomas Schön

**Affiliations:** 1 Department of Immunology and Molecular Biology, University of Gondar, Gondar, Ethiopia; 2 Department of Medical Microbiology, Linköping University, Linköping, Sweden; 3 Department of Medical Laboratory Sciences, Addis Ababa University, Addis Ababa, Ethiopia; 4 Department of Anaesthesia and Intensive Care, Västervik Hospital, Västervik, Sweden; 5 Department of Internal Medicine, University of Gondar, Gondar, Ethiopia; 6 Department of cancer and inflammation, University of Southern Denmark, Odense, Denmark; 7 Department of Infectious diseases, Karolinska Hospital, Stockholm, Sweden; 8 Armauer Hansen Research Institute, Addis Ababa, Ethiopia; 9 Department of Clinical Microbiology and Infectious Diseases, Kalmar County Hospital, Kalmar, Sweden; Ben-Gurion University of the Negev, ISRAEL

## Abstract

**Background:**

The impact of intestinal helminth infection on the clinical presentation and immune response during active tuberculosis (TB) infection is not well characterized. Our aim was to investigate whether asymptomatic intestinal helminth infection alters the clinical signs and symptoms as well as the cell mediated immune responses in patients with active TB.

**Methodology:**

Consecutive, newly diagnosed TB patients and healthy community controls (CCs) were recruited in North-west Ethiopia. TB-score, body mass index and stool samples were analyzed. Cells from HIV-negative TB patients (HIV-/TB) and from CCs were analyzed for regulatory T-cells (Tregs) and cytokine responses using flow cytometry and ELISPOT, respectively.

**Results:**

A significantly higher ratio of helminth co-infection was observed in TB patients without HIV (Helm+/HIV-/TB) compared to HIV negative CCs, (40% (121/306) versus 28% (85/306), p = 0.003). Helm+/HIV-/TB patients showed significantly increased IL-5 secreting cells compared to Helm-/HIV-/TB (37 SFU (IQR:13–103) versus 2 SFU (1–50); p = 0.02, n = 30). Likewise, levels of absolute Tregs (9.4 (3.2–16.7) cells/μl versus 2.4 (1.1–4.0) cells/μl; p = 0.041) and IL-10 secreting cells (65 SFU (7–196) versus 1 SFU (0–31); p = 0.014) were significantly higher in Helm+/HIV-/TB patients compared to Helm-/HIV-/TB patients. In a multivariate analysis, a lower rate of sputum smear positivity for acid fast bacilli, lower body temperature, and eosinophilia were independently associated with helminth infection in TB patients.

**Conclusions:**

Asymptomatic helminth infection is associated with increased regulatory T-cell and Th2-type responses and a lower rate of sputum smear positivity. Further studies are warranted to investigate the clinical and immunological impact of helminth infection in TB patients.

## Author Summary

The effects of helminth infection on chronic infectious diseases such as tuberculosis (TB) merit further characterization. There is limited data regarding the impact of helminth co-infection on clinical and immunological outcomes of TB from clinical field studies in high endemic areas. We tried to address some of these issues in a randomized clinical trial in order to investigate the impact of albendazole treatment on helminth co-infected TB patients. In the present study we focused on the clinical and immunological effects of helminth infection on TB. We found that concomitant asymptomatic helminth infection profoundly affects the immune phenotype of TB patients with a strong leaning towards Th2 types of immune response such as increased regulatory T cells as well as IL-5 and IL-10 secreting cells. Furthermore, helminth co-infection was associated with a significantly lower ratio of sputum smear positivity which correlated to the egg load in helminth positive TB patients. Whether the effect of helminth infection may have an impact on the diagnosis and treatment of active TB remains to be further investigated.

## Introduction

The high prevalence of chronic infectious diseases such as HIV, tuberculosis (TB) and helminth infection has a significant impact on public health in Africa [1]. The majority of TB cases and TB related deaths occur in resource poor countries [2] where TB is the most common life-threatening opportunistic infection in patients with HIV/AIDS [3–4]. In addition, helminths are estimated to infect one third of the world population [5–7] mostly affecting poor, tropical and subtropical areas [8–9].

Symptomatic helminth infection has been shown to be associated with malnutrition measured by low body mass index (BMI) and mid upper arm circumference (MUAC) in children [10]. Helminth infections are widely present in populations where TB and HIV are highly endemic. In high endemic areas of TB such as Ethiopia, helminth co-infection in TB patients has been shown to range between 30–71% [4,11–12].

Protection against TB is strongly associated with enhanced T-helper cell type 1 (Th1) immune responses [13] while susceptibility to the disease is associated with reduced Th1 type responses and enhanced Th2 and regulatory T-cell responses (Tregs) [14]. Evidence is accumulating that chronic helminth infection may affect host immunity in TB [15–17]. The current vaccine against TB, Bacille Calmette Guerin (BCG), has poor efficacy against adult pulmonary TB in sub-Saharan Africa and one reason might be that helminth infection modulates the immune response by increasing Treg activity and thereby attenuating the effects of the vaccine [18].

In a cohort of TB patients in Ethiopia, we previously showed that asymptomatic helminth infection is associated with eosinophilia and elevated IgE levels, supporting the fact that helminth infection affects host immunity during TB [12]. We also recently reported that albendazole treatment of TB patients with helminth infection compared to placebo leads to a reduced level of eosinophilia and IL-10 [19]. In the present study our aim was to compare helminth-positive and helminth-negative TB patients in order to investigate whether asymptomatic helminth infection affects clinical signs and symptoms as well as cell mediated immune responses in patients with active TB.

## Materials and Methods

### Study participants

Consecutive newly diagnosed pulmonary TB patients (15–60 years old) were recruited at the Directly Observed Treatment Short-course (DOTS) clinics located at Gondar University Hospital, Gondar Health Centre and Debark Hospital, Ethiopia. We enrolled smear positive and smear negative pulmonary TB patients at the DOTS clinics after a decision to treat the patients for TB was made by clinicians working in the respective health facilities following clinical, laboratory and chest x-ray examinations according to the WHO based criteria. A patient was considered smear positive when at least one sputum sample was positive for acid fast bacilli (AFB) in the presence of clinical symptoms for active TB. Smear negative TB was defined as clinical symptoms suggestive for TB with three negative sputum smears, radiographic abnormalities consistent with pulmonary TB and a lack of response to a one week course of broad spectrum antibiotic therapy. Exclusion criteria were pregnancy and infectious diseases other than TB and HIV. For purposes of comparison, apparently healthy community controls (CCs) were recruited from the same community. All the CCs were recruited from the blood bank of the Gondar University Hospital. In addition to the routine clinical screening at the blood bank, only blood donors with a TB-score of less than 3 [20] and a normal chest x-ray finding were included as healthy community controls. The data on a subset of TB patients (Helm+/TB) was partly described in a randomized clinical study on deworming presented elsewhere [19] and Helm-/TB patients included in the present study were investigated in an identical way.

### Socio-demographic and clinical characteristics

A structured questionnaire was used to collect socio-demographic and clinical information.The clinical TB-score (from 0 to 13 points) was assessed by measuring signs and symptoms suggestive for TB (cough, haemoptysis, chest pain, dyspnoea, night sweating, tachycardia, temperature >37°C, MUAC and BMI) as previously described [20–21].

### HIV-screening and treatment

Testing for HIV was done at the voluntary counseling and testing clinic and at the DOTS clinic according to the hospital routine with HIV rapid test kits of KHB (HIV 1/2 rapid test strip, China), Stat-Pak (HIV 1/2, Chembio Diagnostics Inc., USA) and Unigold (Trinity Biotech, USA). HIV-positive patients were referred to the HIV clinics for further assessment and free antiretroviral treatment according to Ethiopian HIV/AIDS treatment guideline [22]. All patients were provided Non-Nucleoside Reverse Transcriptase Inhibitors (NNRTI) based regimen where the standard regimen at the time of the study was Efavirenz with Zidovudine and Lamivudine.

### Measurement of CD4^+^ T-cells and eosinophil counts

The CD4^+^T-cells count was analyzed using FACSCount (BD, San Jose, California, USA) available at Gondar and Debark Hospital laboratories. The absolute eosinophil count of peripheral blood was computed in cells/mm3 from the value of total and differential white blood cell counts obtained using Cell Dyn 1800 (Abbot, USA).

### Stool microscopy and acid fast staining of sputum samples

Stool microscopy was done to examine intestinal parasites in samples collected on three consecutive days using direct microscopy and Kato-Katz technique as previously described [23]. The classification of patients into helminth-positive or-negative was based on the results of all three samples together from each patient. One in 10 slides were randomly selected and examined by a second microscopist for quality control. Acid fast bacilli (AFB) load was measured and described using the World Health Organization protocol as: negative (0 AFB/100 oil fields), scanty (1–9 AFB/100 oil fields), + (10–99 AFB/100 oil fields), ++ (1–10 AFB/oil field) and +++ (> 10 AFB/oil field) [24].

### Analysis of regulatory T-cells by flow cytometry

Peripheral blood mononuclear cells, (PBMCs) were isolated by the density gradient separation method using lymphoprep solution as described previously [19,25] and stored at -80°C in FCS containing 10% DMSO. Trypan-blue (Sigma-Aldrich, USA) exclusion was used for determination of cell viability and only samples with viability above 75% were used for experimental assays. PBMCs were stained with CD4-FITC (BD Biosciences), CD25-Percp Cy5.5 (BD Biosciences), and CD127-Alexa 647 (BD Biosciences), followed by fixation/permeabilization using cytofix/cytoperm solution (BD Biosciences) and intracellular staining with monoclonal antibodies (mAbs) against FOXP3- phycoeryhtrin (PE) (BD Biosciences). Tregs were defined as the population of cells being CD4+/CD25hi/CD127low/FOXP3. The total level of FOXP3^+^ CD25^hi^ CD127^lo^ CD4^+^ T cells were determined by multiplying the percentage of these cells in the lymphocyte gate by the number of circulating CD4^+^ T-cells per μl blood.

### ELISPOT analysis of IL-5, IFN-γ and IL-10

Cells (PBMCs) were plated (250,000/well) on PVDF plates (Mabtech, Sweden) coated with a capture mAb specific for human IFN-γ (1-D1K), IL-5 (TRFK5) and IL-10 (9D7) at a concentration of 15μg/ml in PBS. Cells were incubated in a humidified incubator at 37°C in 5% CO_2_ for 24 h with purified protein derivate (PPD, Statens Serum Institute, Denmark) antigens in duplicate using unstimulated and anti-CD3-stimulated cells as negative and positive controls, respectively.

### Ethics statement

Written informed consent was obtained from all study participants and in case of children under 18, from the respective guardians. In addition, children from 15–18 gave assent. The study was approved by the Ethical Review Board of the University of Gondar, Ethiopia and from the Ethical Review Board, Linköping University, Sweden.

### Statistical analysis

Data are presented as median and interquartile ranges. Comparison between groups was done by chi-square test or Fisher´s exact test for discrete variables and Mann-Whitney U test for continuous variables using STATISTICA software (Tulsa, USA). Multiple regression analysis was performed on variables with a P value < 0.1 in the univariate analysis including age, sex and HIV in the model. A P< 0.05 was regarded as statistically significant.

## Results

### Socio-demographic and clinical characteristics

A total of 424 newly and consecutively diagnosed pulmonary TB patients and 306 CCs were included in this study. The overall HIV co-infection rate among TB patients (HIV+/TB) was 28% (118/424), whereas all the CCs were HIV-negative. Seventy percent of the HIV+/TB patients were on anti-retroviral treatment (ART). As the immunological and clinical impact of helminth infection was the main research question and these factors are heavily influenced by HIV, the immunological data were analyzed separately for HIV-/TB patients and the multivariate analysis included HIV in the model. The median age for Helm+/HIV-/TB and Helm-/HIV-/TB patients was 26 (20–35) and 26 (20–37) years, respectively, p = 0.80 (Table 1),with no significant difference in age between CCs with or without helminth infection.

**Table 1:** Socio-demographic characteristics of patients with active tuberculosis infection.

	HIV-/TB patients				
	Helminth-positive	n	Helminth-negative	n	p*
Age, median	26 (20–35)		26 (20–37)		0.801
Sex:					
M	69%	121	59%	185	0.054
F	31%	121	41%	185	0.052
Literate (%)	57%	121	60%	184	0.301
Smoking (%)	15%	120	7%	185	<0.001
Monthly income					
No income	57%	121	65%	183	0.094

*p-value between Helm+/HIV-/TB versus Helm-/HIV-/TB

### Stool microscopy

The prevalence of helminth infection was significantly higher among HIV-/TB patients compared to CCs (40% (121/306) versus 28% (85/306), p = 0.003). The burden of helminth infection was lower at a borderline significant level in HIV+/TB patients compared to HIV-/TB patients (30% (35/118) versus 40% (121/306), p = 0.06 in the univariate analysis). *Ascaris lumbricoides* was the prevailing parasite detected among Helm+/HIV-/TB patients (53%, 63/120) and the CCs (52%, 44/85). No significant differences in the burden of helminth infection were observed among HIV-/TB patients enrolled at the University of Gondar Hospital (42%, 30/71), Gondar Health Center (38%, 53/140) and Debark Hospital (40%, 38/95).

### The clinical impact of helminth infection in patients with active TB

In a multivariate analysis, helminth co-infection was associated with a lower rate of sputum AFB smear positivity and the association was stronger with increasing smear grade (adjusted OR for a smear grade ≥2: 0.43 (95% CI: 0.23–0.79), p = 0.007; Table 2). Furthermore, the mean egg load was significantly lower in patients with a smear grade ≥2 (83±247 eggs, n = 155) compared to patients with a smear grade of 1 (253±900 eggs, n = 122) or sputum smear negative patients (229±607 eggs, n = 103; p = 0.028 Kruskal-Wallis and R = -0.138; p = 0,007 for correlation by the Spearman test). No significant difference was observed between Helm+/TB and Helm-/TB patients for other clinical parameters such as the TB-score (7.5±2.6 versus 7.8±2.5, p = 0.3), BMI or MUAC. A lower body temperature (<37°C), walking bare foot as well as smoking were each independently associated with helminth infection (Table 2).

**Table 2:** Multivariate analysis of the impact of helminth co-infection in patients with active tuberculosis. Laboratory parameters (eosinophilia and sputum smear grade), clinical variables from the TB-score as well as socio-demographic variables and risk factors were analysed in a univariate model where variables with p<0.1 were entered into the multivariate model shown in Table 2. Age, gender and HIV were included in the final model. F = female, M = male.

			Helminths		Univariate		Multivariate	
		N	n	%	OR (95% conf. int.)	p	OR (95% conf. int.)	p
Age	<28	206	80	38.8	1.00		1.00	
	≥28	218	76	34.9	0.84 (0.57–1.25)	0.397	0.67 (0.40–1.11)	0.117
Gender	F	174	60	34.5	1.00		1.00	
	M	250	96	38.4	1.18 (0.79–1.77)	0.411	1.25 (0.74–2.11)	0.117
HIV	No	306	121	39.5	1.00		1.00	
	Yes	118	35	29.7	0.64 (0.41–1.02)	0.060	0.79 (0.45–1.39)	0.413
Walking bare foot	No	307	118	38.4	1.00		1.00	
	Yes	118	30	25.4	1.68 (1.11–2.54)	0.013	1.84 (1.11–3.06)	0.019
Smoking	No	374	129	34.5	1.00		1.00	
	Yes	48	25	52.1	2.06 (1.12–3.79)	0.019	3.20 (1.42–7.19)	0.005
Sputum smear grade	0 (negative)	114	53	46.5	1.00		1.00	
	1	131	47	35.9	0.73 (0.57–0.94)		0.65 (0.48–0.89)	
	≥2	168	53	31.5	0.54 (0.33–0.88)	0.013	0.43 (0.23–0.79)	0.007
Eosinophilia (>300 cells/μl)	No	161	58	36.0	1.00		1.00	
	Yes	156	82	52.6	1.97 (1.25–3.09)	0.003	1.98 (1.22–3.22)	0.006
Body temperature > 37°C	No	141	60	42.6	1.00		1.00	
	Yes	283	96	33.9	0.69 (0.46–1.05)	0.084	0.62 (0.37–1.04)	0.067

### Helminth infection is associated with increased regulatory T-cell frequency and Th2-type response in TB patients

**Fig 1:**
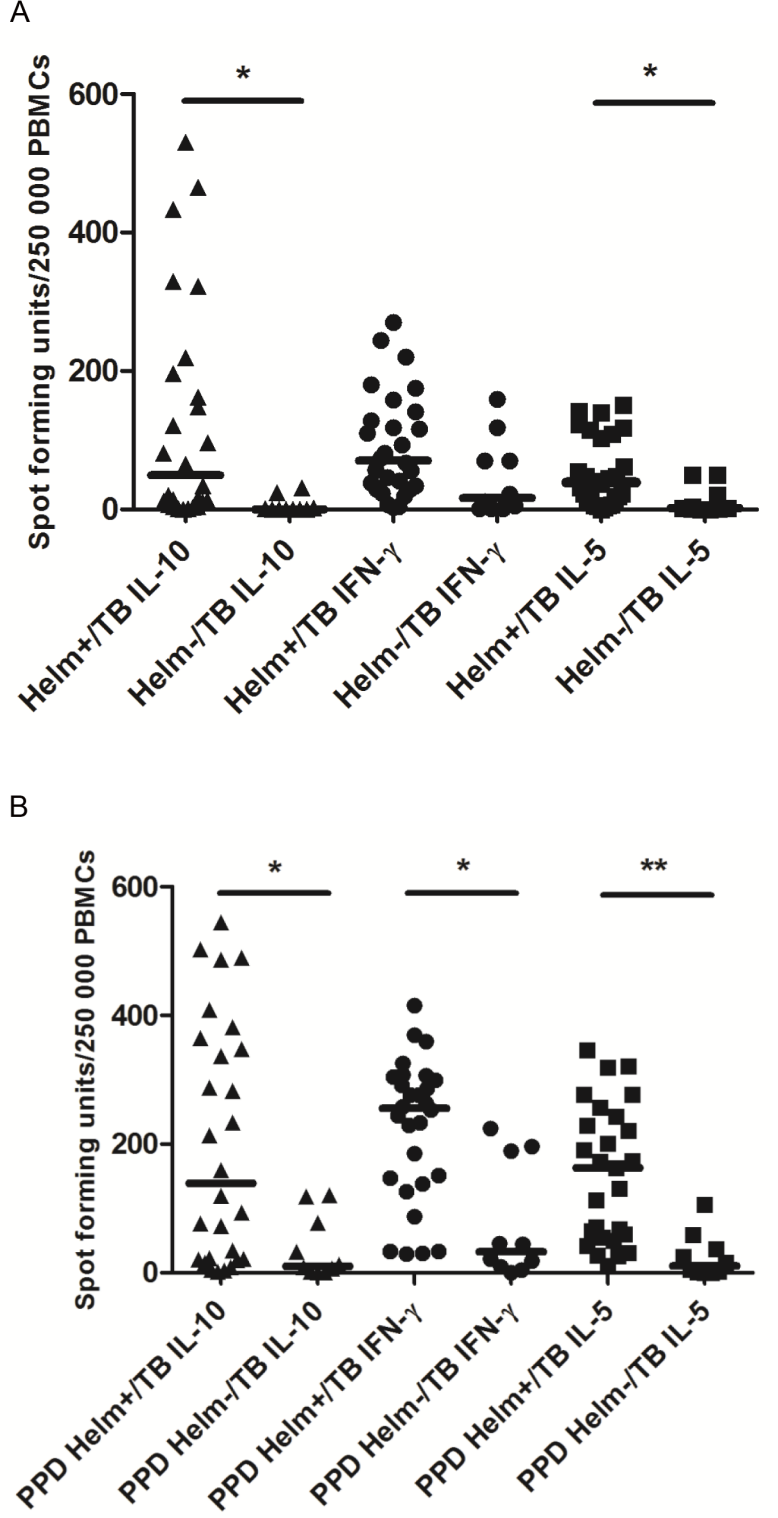
A-B. Distribution of IFN-gamma, IL-5 and IL-10 in HIV-/tuberculosis (TB) patients with and without helminth (Helm) infection analyzed by ELISPOT. **A.** Unstimulated cells. **B.** PPD-stimulated cells. Vertical bars lines represent the median level of spot forming units (SFU) per 250 000 peripheral blood mononuclear cells (PBMCs). *p<0.05 and **p<0.01.

In the multivariate analysis, eosinophilia (>300 cells/mm^3^) was independently associated with helminth infection in TB patients (adjusted OR: 1.98 (95% CI: 1.2–3.2), p = 0.007) (Table 2). We analyzed IFN-γ, IL-5 and IL-10 secreting cells from a subset of 30 HIV negative TB patients and 56 CCs with and without helminth infection. Significantly increased median levels of IL-5 and IL-10 secreting cells measured in the ELISPOT-assay by spot forming units (SFU) were observed in unstimulated cells from Helm+/HIV-/TB patients compared with Helm-/HIV-/TB patients (IL-5: 37 SFU(IQR:13–103) versus 2 SFU(1–50); p = 0.02) and (IL-10: 65 SFU(7–196) versus 1 SFU (0–31); p = 0.014), respectively (Fig 1A).Similarly, a significant increment in IL-5 (147 SFU (52–236) versus 16 SFU (2–59); p = 0.0017) as well as IL-10 secreting cells (119 SFU (19–347) versus 32 (1–118); p = 0.046) were observed following PPD stimulation among Helm+/HIV-/TB patients (Fig 1B). There was no difference in the levels of IFN-γ secreting cells between the two groups for unstimulated cells (57 SFU (29–118) versus 67 SFU (1–118); p = 0.45) but a significant difference in PPD-stimulated cells (244 SFU (126–291 vs 45 SFU (9–196), p = 0.027). The absolute levels of Tregs were also increased in Helm+/HIV-/TB compared to Helm-/HIV-/TB patients (9.4 (3.2–16.7) cells/μl versus 2.4 (1.1–4.0) cells/μl; p = 0.041). In the healthy controls (CCs), no significant differences were observed in IFN-γ, IL-5, IL-10 secreting cells nor in absolute levels of Tregs among Helm+/CCs versus Helm-/CCs, respectively (S1A and S1B Fig).

## Discussion

The main finding of this study is that during active TB, helminth infection is associated with increased regulatory T-cell and Th2-type responses. Additionally, helminth infection is significantly correlated to a reduced rate of sputum smear positivity with a correlation between an increasing sputum smear grade and a decreasing egg load.

We found increased level of IL-10 associated with helminth infection in TB patients as compared to helminth negative TB patients. This is in line with a recent report where we observed that IL-10 was significantly reduced following albendazole treatment of helminth-positive TB patients compared to placebo [19]. In HIV-patients without TB, a previous clinical trial also showed that albendazole treatment induced a decline in serum IL-10 levels [26]. Altogether, this indicates a role for IL-10 in the helminth induced immunomodulation. Anti-helminthic treatment in TB patients may therefore decrease worm burden, leading to impairment of the IL-10 production and improved cellular immunity to TB which could have potential clinical benefits.

Recent results indicate that increased activity of Tregs and regulatory cytokines such as IL-10 may contribute to pathogen persistence by modulating the host immune response in infectious diseases such as TB [27]. In animal models, it has been shown that Tregs are associated with helminth infection [28]. Regulatory T-cells are common in the gut which is the usual site of infection for helminths [29]. The Th2-dominance in many infections such as TB is maintained by IL-10- and TGF-β mediated suppression of competing Th1 and Th17-cell populations [18]. This has been shown in a mouse filariasis model using *L*. *sigmodontis* where the chronic phase of infection is marked by T-cell anergy, loss of proliferative responses to parasite antigen, reductions in effector cytokine levels, and elevated expression of inhibitory cytokines and cells such as IL-10 and Tregs [30]. In human filarial and ascaris infections, the presence of helminths correlate with both increased production of IL-10 [31], and generalized T-cell hypo-responsiveness [32]. In addition, the observed epidemiological association of increased prevalence of helminth infection with a lower ratio of allergic manifestations and autoimmune inflammatory conditions in humans is linked to an attenuated immune response by Tregs and IL-10 [33–35]. Altogether, these experimental and clinical data suggest an immunosuppressive role for increased regulatory T-cell and IL-10 activity [26] which is in line with our present findings.

Regarding the clinical impact of helminth infection in TB patients, we found a lower sputum smear rate and lower body temperature among Helm+/HIV-/TB patients. Interestingly, helminth negative TB patients showed an association to increasing sputum smear grade and among helminth positive TB patients, there was a correlation between higher sputum smear grades and lower helminth egg loads. One possible explanation might be that the higher level of Tregs and increased levels of the regulatory cytokine, IL-10, could suppress the inflammatory and Th1-associated immune responses leading to reduced cavity formation and consequently a reduced sputum smear load. A similar pattern has been reported in HIV/TB co-infection where HIV was associated to a reduction in CD4^+^ counts together with a reduced sputum smear positivity rate compared to HIV-/TB patients [36–38]. In HIV co-infected TB patients, it is known that the inflammatory infiltrate is less well organized with reduced cavity formation along with increasing immunosuppression. Thus, the immunological impact of HIV and helminth co-infection in TB may result in reduced sensitivity of the sputum smear examination, a diagnostic method still widely used in high endemic areas.

An association between increased prevalence of intestinal helminth infection and pulmonary TB has been repeatedly reported from the present study area [4,11–12]. Consistent with our previous finding in another cohort of TB patients from the same area [12], we verified again that close to 40% of TB patients are asymptomatically infected with intestinal parasites.

The evaluation of helminth infection was based on the examination of three consecutive stool samples, by both direct and the Kato-Katz method. As limitation of this study, this method could miss low-grade helminth infection. Moreover, the immunological impact of helminths may be species dependent but apart from *Ascaris lumbricoides*, this correlation could not be evaluated within the present study due to a limited samples size. Furthermore, the TB diagnosis was based on the clinician’s intention to treat a patient with anti-TB drugs based on clinical, sputum smear, and chest x-ray examinations according to the WHO based criteria for smear positive and smear negative TB and not by sputum culture confirmation. The level of non-tuberculosis mycobacteria is very low and was not detected in a previous study of smear positive TB patients from the area [39] which is consistent with other reports from Ethiopia showing that NTMs are found at a frequency less than 2% among smear positive TB patients [40].

In summary, we show that helminth infection is significantly associated with increased regulatory T-cell activity, an increased Th2 type response, as well as reduced rate of sputum smear positivity in patients with active TB in a high endemic area for both TB and helminth infection. These results may have implications for diagnosis and immunotherapy in patients from areas where both infections are endemic.

## Supporting Information

S1 Checklist STROBE Checklist. (DOC)

S1 Fig A-B. Distribution of IFN-gamma, IL-5 and IL-10 in HIV negative healthy community controls (CCs) with and without helminth (Helm) infection analyzed by ELISPOT. A. Unstimulated cells. B. PPD-stimulated cells. Vertical bars lines represent the median level of spot forming units (SFU) per 250 000 peripheral blood mononuclear cells (PBMCs).
(RTF)
