# Supplementary material for: Asymptomatic Helminth Infection in Active Tuberculosis Is Associated with Increased Regulatory and Th-2 Responses and a Lower Sputum Smear Positivity
Source: PLoS Negl Trop Dis. 2015 Aug 6;9(8):e0003994. doi: 10.1371/journal.pntd.0003994 (PMC4527760; doi:10.1371/journal.pntd.0003994)
Supplement: S1 Fig — A. Unstimulated cells. B. PPD-stimulated cells. Vertical bars lines represent the median level of spot forming units (SFU) per 250 000 peripheral blood mononuclear cells (PBMCs). (RTF) [file pntd.0003994.s002.rtf]

Supporting Information Figures
Fig. S1a


Fig. S1b
